# Comparing rumen fluid collection methods on fermentation profile and microbial population in lactating dairy cows

**DOI:** 10.3168/jdsc.2024-0566

**Published:** 2024-07-14

**Authors:** N. Pathak, H. Guan, P. Fan, H. Sultana, K. Arriola, A. Oyebade, C. Nino de Guzman, M. Malekkhahi, K.C. Jeong, D. Vyas

**Affiliations:** Department of Animal Science, University of Florida, Gainesville, FL 32611

## Abstract

The objective of this study was to compare fermentation profile and microbial diversity from rumen samples collected using a rumen cannula (RC) or stomach tube (ST) in lactating dairy cows. Three ruminally cannulated lactating dairy cows were used in a 3 × 3 Latin square design. The experimental period was 28 d and rumen fluid was collected 4 h after feeding on d 22 and 26 of each experimental period. Treatments were rumen fluid collected from the ST or RC. The pH of rumen contents was measured immediately after fluid collection and samples were analyzed for VFA, ammonia-N (NH_3_-N) concentration, and microbiome composition. Rumen pH was greater for ST compared with RC (6.88 vs. 6.25). However, NH_3_-N (15.2 vs. 10.6 mg/dL) and total VFA (121.8 vs. 95.5 m*M*) were greater for RC compared with ST. The rumen fluid collection methods had no effects on the molar proportion of acetate and propionate; however, the acetate-to-propionate ratio tended to increase with ST compared with RC. The majority of sequences obtained with microbiome analysis belonged to *Bacteroidetes*, *Firmicutes*, and *Cyanobacteria*, regardless of the rumen fluid collection method. However, the abundance of *Bacteroidetes* was greater and the abundance of *Firmicutes* was lower for ST compared with RC. No effects were observed on the abundance of phylum *Cyanobacteria*. The rumen fluid collection methods had no effects on Chao1 and Shannon index. In conclusion, the molar proportion of individual VFA, Chao1, and Shannon index were similar, whereas rumen pH, NH_3_-N, and total VFA were affected by the rumen fluid collection method.

## Highlights

• ST had higher pH and lower NH_3_-N and total VFA compared with RC.
• RC had lower Bacteroidetes and higher *Firmicutes* compared with ST.
• Chao1 and Shannon index were similar between RC and ST.

## Graphical Abstract

**Figure:**
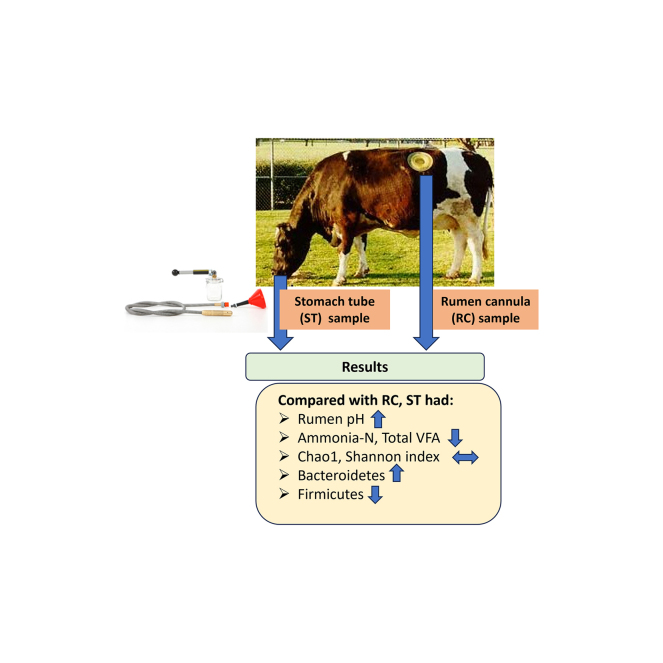
**Summary:** This study aimed to assess the impact of different rumen fluid collection methods on fermentation profile and microbial diversity in lactating dairy cows. Utilizing 3 cows in a 3 × 3 Latin square design, rumen contents were collected via a stomach tube (ST) and a rumen cannula (RC). Samples were analyzed for rumen pH, volatile fatty acids (VFA), ammonia-N (NH_3_-N) concentration, and microbiome composition. Stomach tube samples had greater pH and lower NH_3_-N and total VFA concentrations. Both methods yielded similar molar proportions of acetate (A) and propionate (P), although the A:P ratio tended to increase with ST. Stomach tube samples had a greater abundance of *Bacteroidetes* and lower *Firmicutes* compared with RC, with no differences in Cyanobacteria abundance. Chao1 and Shannon indexes were similar, indicating similar microbial richness and diversity regardless of the collection method.

An accurate understanding of rumen fermentation and ruminal microbiome (Hook et al., 2009; Lodge-Ivey et al., 2009; Terré et al., 2013) is crucial to develop dietary strategies for improving production efficiency and reducing the environmental impact of ruminant production systems (Terré et al., 2013). Rumen sampling techniques can affect both fermentation parameters and microbiome analysis. Rumen cannulation (**RC**) is considered the reference method for rumen fluid collection because of the ease of collecting representative samples (Beharka et al., 1998); however, cannulation requires surgical alteration and is considered an invasive method that may not be broadly applicable. Less invasive alternatives like pumping ruminal fluid using an oral stomach tube (**ST**) have also been used for collecting rumen fluid samples (Abdelgadir et al., 1996; Coverdale et al., 2004); however, samples collected using ST are more susceptible to saliva contamination (Terré et al., 2013). The objectives of this study were to compare rumen fermentation parameters and microbial population of rumen fluid collected using RC or ST. We hypothesized that the fermentation profile including rumen pH and VFA will be different; however, the fermentation pattern and abundance of dominant microbes will be comparable between both techniques.

The ruminal fluid collection protocol was approved by the University of Florida Institutional Animal Care and Use Committee (protocol number: 202300000409). Three rumen-cannulated lactating multiparous Holstein cows with an average (mean ± SD) 165 ± 63 DIM, 25 ± 9 kg milk/d, 880 ± 54 kg of BW were used in a 3 × 3 Latin square design study with a 28-d experimental period. Each period had a 21-d adaptation phase followed by 7 d of rumen fluid collection period. Treatments were (1) rumen fluid collected using ST on d 22 and 26 of each experimental period and (2) rumen fluid collected from RC on d 22 and 26 of each experimental period. Experimental animals were offered diets formulated based on NRC (2001) to meet or exceed the nutrient requirements of lactating dairy cows producing 42 kg/d of milk, with 3.80% milk fat and 3.20% milk protein, and consuming 26 kg/d DMI (NRC, 2001). Animals had free access to water. Total mixed ration was composed of corn silage (47%), ground corn (18.9%), soybean meal (15.15%), citrus pulp (4.71%), whole cottonseed (7.37%), fat supplement (1.68%; Palmit-80, Global Agri Trade Corporation, Long Beach, CA), and a mineral-vitamin premix (5.21%) containing 7.44% Ca, 1.6% P, 2.52% Mg, 0.21% K, 0.44% S, 8.13% Na, 3.30% Cl, 1,450 mg/kg Zn, 220 mg/kg Cu, 7.33 mg/kg Se, 273.22 kIU/kg vitamin A, 69.95 kIU/kg vitamin D, and 546.44 IU/kg vitamin E. The analyzed chemical composition (% of DM) of TMR constitutes CP (16.4%), amylase- and sodium sulfite-treated NDF corrected for ash residue (**aNDFom**; 28%), starch (28.6%), and NFC (42.5%).

Ruminal fluid (∼200 mL) was collected 4 h after the morning feeding using an orally administered ST connected to a vacuum pump (Ruminator; profs-products.com, Wittibreut, Bayern, Germany). Approximately 200 mL of rumen fluid was collected after discarding the initial 200 mL to minimize saliva contamination. Before straining the ST rumen samples through cheesecloth, samples were visually examined to ensure the absence of saliva contamination. Rumen fluid from RC was collected immediately after ST sample collection and contents were sampled from cranial, caudal, dorsal, and ventral aspects of the rumen. Both RC and ST samples were filtered through 4 layers of cheese cloth and rumen fluid pH was measured with a pH meter (Accumet AB15, Fisher Scientific, Hampton, NH). Rumen fluid (∼40 mL) from each sample was stored at −80°C for analysis of microbial diversity and abundance. Another set of 40-mL samples was acidified with 400 μL of 50% H_2_SO_4_. Acidified rumen samples were centrifuged at 11,500 × *g* for 20 min at 4°C and the supernatant was stored at −80°C until analyzed for VFA and NH_3_-N concentration.

Total and individual VFA, including acetate, propionate, butyrate, isobutyrate, and isovalerate, were measured using an HPLC (FL 7485, Hitachi, Tokyo, Japan) according to the method of Muck and Dickerson (1988). The column (Aminex HPX-87H, Bio-Rad Laboratories, Hercules, CA) used a 0.015 *M* H_2_SO_4_ mobile phase and a flow rate set at 0.70 mL/min at 45°C and was connected to a UV detector (Spectroflow 757, ABI Analytical Kratos Division, Ramsey, NJ) set at 210 nm. Ammonia-N analysis was performed using the phenol-hypochlorite method (Broderick and Kang, 1980).

For microbiome analysis, rumen fluid samples were thawed, and DNA was extracted and purified using the PowerLyzer PowerSoil DNA isolation kit (MOBIO Laboratories Inc., Carlsbad, CA) with bead beating, following the manufacturer's protocol. Briefly, bead beating steps were performed for 3 min (Bullet159 Blender Storm 24, Next Advance, Averill Park, NY) using a 0.1-mm bead to homogenize the suspension and mechanically disrupt the bacterial cells. Samples were then kept for 15 min at 70°C without beating and another bead beating protocol was repeated for 3 min. The DNA concentration and purity were measured using a Nanodrop ND-1000 (Thermo Fisher Scientific, Waltham, MA). The mean DNA concentration of samples was 68.65 ng/μL, and the absorbance (**A**) ratio at 260 and 280 nm (A_260_/A_280_) ratio was between 1.75 and 1.88.

The V4 region of the 16S rRNA gene was amplified using the dual-index primers (Kozich et al., 2013), purified and normalized using the SequalPrep plate normalization kit (Invitrogen, United States), and sequenced on the MiSeq platform (2 × 250 bp). The sequencing product was analyzed using agarose (0.7%) gel electrophoresis. Raw sequencing reads were obtained from the Illumina BaseSpace website and analyzed with the Quantitative Insights into the Microbial Ecology 2 (QIIME 2) pipeline. The sequence quality control was performed with the Divisive Amplicon Denoising Algorithm (DADA2) pipeline implemented in QIIME 2, including steps for filtering low quality reads, denoising reads, merging the paired-end reads, and removing chimeric reads. The sequencing depth was normalized to 21,250 sequences per sample. Both α diversity (Chao1 and Shannon indices) and β diversity (weighted UniFrac distance) were analyzed using the QIIME2 software package with a script core-metrics-phylogenetic method. Differences between weighted UniFrac distances were analyzed using a permutational multivariate ANOVA (PERMANOVA) with the β-group-significance command in QIIME 2 pipeline. All amplicon sequencing variants were classified into the bacterial taxonomy using the q2-feature-classifier plugin of QIIME 2 and the SILVA 132 database. The relative abundance of bacterial taxa at 6-level taxonomic classification (phylum, class, order, family, genus, and species) was obtained using the q2-feature-classifier plugin.

Data were analyzed using PROC GLIMMIX procedure SAS version 9.4 (SAS Institute Inc., Cary, NC). The statistical model included fixed effects of technique for rumen fluid collection, period, and interaction. The normal distribution of residuals was evaluated using the UNIVARIATE procedure of SAS. The Shapiro-Wilk statistic was used to determine if the residuals were normally distributed (value ≥0.10). Significance was declared at *P* ≤ 0.05, and tendencies were reported if *P* values were between 0.05 and 0.10.

Rumen pH, NH_3_-N, and VFA proportions are presented in Table 1. Rumen pH was greater (6.88 vs. 6.25; *P* < 0.01), whereas total VFA (95.5 vs. 121.8 m*M*; *P* < 0.01) and NH_3_-N concentration (10.6 vs. 15.2 mg/dL; *P* = 0.01) were lower in ST compared with RC samples. However, individual VFA molar proportions were similar between ST and RC methods. The differences in the fermentation profile between ST and RC samples observed in this study are in agreement with previous findings (Shen et al., 2012; Terré et al., 2013). Duffield et al. (2004) suggested that saliva contamination with ST samples may increase pH and subsequently influence fermentation profile when compared with RC samples; however, the first aliquot of rumen fluid samples collected from ST was discarded in the present study and we speculate that differences in pH between ST and RC may not be completely attributed to saliva contamination. Shen et al. (2012) reported that sampling depth may influence differences in pH between ST and RC samples. The fermentation variables in rumen samples collected from ST inserted at a depth of 180 cm were different from RC samples; however, the differences disappeared when rumen samples were collected with ST inserted at a depth of 200 cm (Shen et al., 2012). In addition, Larsen et al. (2020) reported that differences in fermentation variables between RC and ST may be attributed to sampling location since ST may not pass beyond cranial or dorsal rumen due to firm digesta, whereas RC samples are more representative of all parts of the rumen. Wang et al. (2016) reported numerically lower VFA and greater pH when rumen contents were sampled from the cranial dorsal compared with cranial and caudal ventral sac probably due to saliva flow to the rumen because of its close proximity to the esophagus. Overall, findings from previous studies further support our speculation that ST samples are more representative of the dorsal sac of the rumen. Contrary to the lack of differences in individual VFA profile, the acetate-to-propionate ratio tended to be greater for ST compared with RC samples (3.01 vs. 2.85; *P* = 0.08). The molar proportion of acetate was numerically greater (58.7 vs. 57.8; *P* = 0.20), whereas propionate proportion was numerically lower (19.6 vs. 20.6; *P* = 0.16) within ST samples and contributes toward the tendency for greater acetate-to-propionate ratio. Although it is difficult to explain the results observed on greater acetate-to-propionate ratio considering minimal changes were observed in individual VFA molar proportion, we can speculate that the increased abundance of *Bacteroidetes* in ST samples may have increased plant cell wall–degrading activities resulting in a higher acetate-to-propionate ratio.

**Table 1:** Effects of technique of rumen content collection on fermentation characteristics

Item	Technique^1^		SEM	*P*-value	
	ST	RC		Technique	Period
Rumen pH	6.88	6.25	0.16	<0.01	0.88
NH_3_-N, mg/dL	10.6	15.2	1.16	0.01	<0.01
Total VFA, m*M*	95.5	121.8	5.31	<0.01	<0.01
Individual VFA, mol/100 mol					
Acetic acid	58.7	57.8	1.02	0.20	0.09
Propionic acid	19.6	20.6	1.03	0.16	0.80
Isobutyric acid	3.37	3.80	0.79	0.64	0.28
Butyric acid	13.9	13.4	0.44	0.36	0.11
Isovaleric acid	2.83	2.79	0.22	0.87	0.64
Valeric acid	1.64	1.63	0.21	0.98	0.18
Acetate-to-propionate ratio	3.01	2.85	0.18	0.08	0.77

1 Stomach tube (ST) or rumen cannula (RC): With 3 × 3 Latin square design, we had 9 observations for each technique after the end of 3 experimental periods.

Although no period effect was observed on pH of the rumen contents, total VFA was greater in the first and second period compared with the third period (*P* < 0.01). Similarly, the NH_3_-N concentration was lowest in the third period, whereas the maximum concentration was observed in the second period (*P* < 0.01). No period effects were observed on the molar proportion of individual VFA except for a tendency of greater acetate proportion in the third period of this study (*P* = 0.08).

Bacterial community composition in the rumen is dependent on the specific fraction of rumen contents being analyzed with notable differences observed between the solid, liquid, and whole digesta, which further underscores the importance of considering the heterogeneity within the rumen environment when studying microbial populations (de Assis Lage et al., 2020; Indugu et al., 2021). In the current study, the rumen bacterial communities obtained from next-generation sequencing (**NGS**) analysis showed that *Bacteroidetes*, *Firmicutes*, and *Cyanobacteria* were the most abundant phyla in both ST and RC samples, making up to >90% of the whole bacterial community (Table 1) and the results are in agreement with previous studies (Wallace, 2008; Stewart et al., 2018) using both culture-based and NGS methods. Strained rumen contents used for microbial analysis in this study are more representative of liquid samples because of the sampling procedure used in this study. de Assis Lage et al. (2020) observed greater species richness in the liquid fraction of rumen contents collected using ST compared with RC; however, no differences were observed on Shannon index. Lodge-Ivey et al. (2009) also reported similar Shannon index between ST and RC samples collected from sheep and goat. In the present study, Chao1 indices, representative of species richness, were numerically greater for RC samples compared with ST samples (591 vs. 554, *P* = 0.14); however; Shannon index, representative of species evenness, was similar in both ST and RC sample collection methods (*P* = 0.21). Bacterial community composition, specifically relative abundance of bacterial population, was different in the liquid fraction of rumen contents collected using RC and ST methods (de Assis Lage et al., 2020) and the results are in agreement with the present study. On the contrary, Song et al. (2018) observed no difference in microbial profiles between RC and ST samples collected from Hanwoo steers fed high concentrate diets. We observed 11% greater (*P* < 0.01) relative abundance of *Bacteroidetes* phylum in ST samples; however; RC samples had higher relative abundance of *Firmicutes* (*P* < 0.01), *Spirochaetes* (*P* = 0.04), and *Actinobacteria* (*P* = 0.02) by 15.5%, 21.8%, and 22.7%, respectively, compared with ST samples (Table 2). Beta diversity, measured by weighted UniFrac distance, differed between RC and ST samples (*P* = 0.001; Figure 1). At the genus level, the rumen fluid collected from ST had 23% greater abundance of *Prevotella* compared with RC, whereas *Christensenellaceae R-7*, *Ruminococcaceae NK4A214*, and *Treponema* were the most abundant in RC samples (Table 2). In both RC and ST samples, *Prevotella* emerged as the most dominant genus and these findings are in agreement with a previous study (Bowen et al., 2018). Sbardellati et al. (2020) reported that *Prevotellaceae* and *Prevotella* are predominant in the microbial population in the rumen of cattle, regardless of ruminal locations. However, a greater abundance of *Prevotellaceae* was observed in the caudodorsal blind sac compared with the cranial, ventral, and caudodorsal blind sac in the rumen (Sbardellati et al., 2020). Ruminal *Prevotella* have diverse functions including hemicellulolytic and proteolytic activities (Matsui et al., 2000) and greater abundance within microbial communities of the dorsal sac may reflect their metabolic flexibility and overall dominance in the rumen environment. Bowen et al. (2018) noted a higher prevalence of *Prevotella* in the liquid phase of the rumen, which could lead to an increased abundance of *Bacteroidetes* in the liquid fraction of the rumen samples (Jewell et al., 2015). It is plausible to speculate that ST samples might have disproportionately represented the liquid phase of the rumen contents more than RC samples; however, the greater abundance of *Prevotellaceae* is more likely due to ST samples representing the dorsal sac of the rumen. The lower abundance of *Lachnospiraceae* in the ST samples further supports our speculation that a greater proportion of dorsal sac rumen contents is associated with this sampling method because Sbardellati et al. (2020) observed similar differences when rumen samples from caudodorsal blind sac were compared with the ventral sac. The lower abundance of *Ruminococcaceae* in ST samples were not in agreement with previous findings. Paz et al. (2016) did not observe an effect of rumen fluid collection methods (ST vs. RC) on *Ruminococcaceae* using 16S rRNA gene sequencing analysis. Similarly, Sbardellati et al. (2020) observed no differences in the abundance of *Ruminococcaceae* between different sampling locations in the rumen.

**Table 2:** Effects of technique of rumen content collection on microbial diversity and the relative abundance of dominant bacterial phyla, family, and genus

Item	Technique^1^		SEM	*P*-value	
	ST	RC		Technique	Period
Diversity index					
Chao1	554	591	18.6	0.14	0.26
Shannon	8.62	8.72	0.06	0.21	0.70
Bacterial phyla					
*Bacteroidetes*	60.5	53.8	1.11	<0.01	0.43
*Firmicutes*	25.0	29.6	1.04	<0.01	0.26
*Proteobacteria*	1.07	1.04	0.22	0.91	0.60
*Spirochaetes*	4.72	6.04	0.87	0.04	0.08
*Cyanobacteria*	3.31	2.83	0.84	0.32	0.14
*Actinobacteria*	0.75	1.10	0.26	0.02	0.12
*Fibrobacteres*	0.77	0.81	0.18	0.88	0.74
Family					
*Prevotellaceae*	50.6	39.2	1.14	<0.01	0.02
*Ruminococcaceae*	6.88	9.03	0.75	0.03	0.03
*Veillonellaceae*	2.69	1.93	0.30	0.02	0.07
*Rikenellaceae*	3.23	3.89	0.48	0.01	0.13
*Lachnospiraceae*	5.71	6.95	0.86	0.04	0.66
*Spirochaetaceae*	4.70	6.02	0.90	0.04	0.08
*Acidaminococcaceae*	6.88	7.54	0.98	0.60	0.23
Genus					
*Prevotella*	36.7	28.1	1.14	<0.01	0.04
*Prevotellaceae UCG-001*	4.67	4.16	0.35	0.21	0.48
*Prevotellaceae UCG-003*	2.76	2.69	0.35	0.74	0.21
*Rikenellaceae family Alistipes*	3.11	3.54	0.51	0.09	0.31
*Christensenellaceae R-7 group*	1.48	2.33	0.21	<0.01	0.07
*Ruminococcaceae NK4A214 group*	2.22	3.35	0.26	<0.01	0.07
*Succiniclasticum*	6.87	7.53	1.00	0.60	0.22
*Treponema*	4.60	5.85	0.84	<0.01	0.08

1 Stomach tube (ST) or rumen cannula (RC): With 3 × 3 Latin square design, we had 9 observations for each technique after the end of 3 experimental periods.

**Figure 1:**
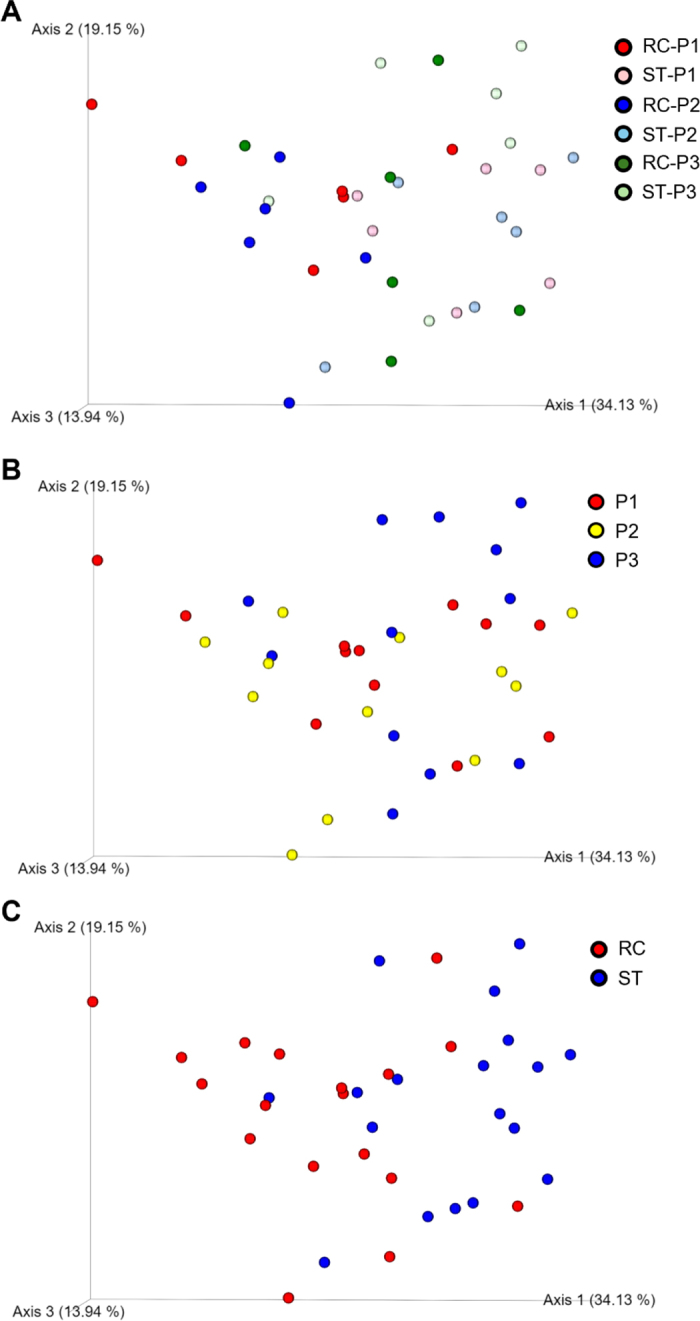
Differences in weighted UniFrac distance were analyzed by permutational multivariate ANOVA (PERMANOVA). (A) Treatment and period effects, *P* = 0.52; (B) is treatment effect, *P* = 0.001, while (C) is period effect, *P* = 0.489; n = 36. RC = rumen cannula; ST = stomach tube; P1 = period 1; P2 = period 2; P3 = period 3.

In conclusion, we observed differences in rumen pH, NH_3_-N, and total VFA concentrations in rumen samples collected using ST and RC methods as ST samples exhibited greater pH and lower NH_3_-N and total VFA concentrations compared with RC samples. Both methods yielded similar molar proportions of acetate and propionate, although the acetate-to-propionate ratio tended to be greater in ST samples. Both species richness and evenness were comparable between the methods, but the relative abundance of specific bacterial phyla varied. Stomach tube samples had a greater abundance of *Bacteroidetes* and a lower abundance of *Firmicutes* compared with RC samples. We speculate that differences in fermentation profile between 2 methods are primarily due to sampling location differences and not due to saliva contamination because precautions were taken to avoid saliva from ST samples. Overall, although the RC method for collecting rumen samples is still preferred for its accuracy and ability to collect representative samples, the ST method can be a feasible alternative, especially for comparing treatment effects on molar proportions of individual VFA and richness and evenness of microbial species. However, researchers using the ST method should be aware of potential deviations in rumen fermentation and the microbial population.

## References

[bib1] Abdelgadir I.E.O., Morrill J.L., Higgins J.J. (1996). Effect of roasted soybeans and corn on performance and ruminal and blood metabolites of dairy calves. J. Dairy Sci..

[bib2] Beharka A.A., Nagaraja T.G., Morrill J.L., Kennedy G.A., Klemm R.D. (1998). Effects of form of the diet on anatomical, microbial, and fermentative development of the rumen of neonatal calves. J. Dairy Sci..

[bib3] Bowen J.M., McCabe M.S., Lister S.J., Cormican P., Dewhurst R.J. (2018). Evaluation of microbial communities associated with the liquid and solid phases of the rumen of cattle offered a diet of perennial ryegrass or white clover. Front. Microbiol..

[bib4] Broderick G.A., Kang J.H. (1980). Automated simultaneous determination of ammonia and total amino acids in ruminal fluid and in vitro media. J. Dairy Sci..

[bib5] Coverdale J.A., Tyler H.D., Quigley J.D., Brumm J.A. (2004). Effect of various levels of forage and form of diet on rumen development and growth in calves. J. Dairy Sci..

[bib6] de Assis Lage C.F., Räisänen S.E., Melgar A., Nedelkov K., Chen X., Oh J., Fetter M.E., Indugu N., Bender J.S., Vecchiarelli B., Hennessy M.L., Pitta D., Hristov A.N. (2020). Comparison of two sampling techniques for evaluating ruminal fermentation and microbiota in the planktonic phase of rumen digesta in dairy cows. Front. Microbiol..

[bib7] Duffield T., Plaizier J.C., Fairfield A., Bagg R., Vessie G., Dick P., Wilson J., Aramini J., McBride B. (2004). Comparison of techniques for measurement of rumen pH in lactating dairy cows. J. Dairy Sci..

[bib8] Hook S.E., Northwood K.S., Wright A.D., McBride B.W. (2009). Long-term monensin supplementation does not significantly affect the quantity or diversity of methanogens in the rumen of the lactating dairy cow. Appl. Environ. Microbiol..

[bib9] Indugu N., Hennessy M., Kaplan-Shabtai V.S., de Assis Lage C.F., Räisänen S.E., Melgar A., Nedelkov K., Chen X., Oh J., Vecchiarelli B., Bender J.S., Hristov A.N., Pitta D.W. (2021). Comparing noninvasive sampling techniques with standard cannula sampling method for ruminal microbial analysis. JDS Commun..

[bib10] Jewell K.A., McCormick C.A., Odt C.L., Weimer P.J., Suen G. (2015). Ruminal bacterial community composition in dairy cows is dynamic over the course of two lactations and correlates with feed efficiency. Appl. Environ. Microbiol..

[bib11] Kozich J.J., Westcott S.L., Baxter N.T., Highlander S.K., Schloss P.D. (2013). Development of a dual-index sequencing strategy and curation pipeline for analyzing amplicon sequence data on the MiSeq Illumina sequencing platform. Appl. Environ. Microbiol..

[bib12] Larsen M., Hansen N.P., Weisbjerg M.R., Lund P. (2020). Technical note: Evaluation of the ororuminal FLORA sampling device for rumen fluid sampling in intact cattle. J. Dairy Sci..

[bib13] Lodge-Ivey S.L., Browne-Silva J., Horvath M.B. (2009). Technical note: Bacterial diversity and fermentation end products in rumen fluid samples collected via oral lavage or rumen cannula. J. Anim. Sci..

[bib14] Matsui H., Ogata K., Tajima K., Nakamura M., Nagamine T., Aminov R.I., Benno Y. (2000). Phenotypic characterization of polysaccharidases produced by four *Prevotella* type strains. Curr. Microbiol..

[bib15] Muck R.E., Dickerson J.T. (1988). Storage temperature effects on proteolysis in alfalfa silage. Trans. ASAE.

[bib16] NRC (2001).

[bib17] Paz H.A., Anderson C.L., Muller M.J., Kononoff P.J., Fernando S.C. (2016). Rumen bacterial community composition in Holstein and Jersey cows is different under same dietary condition and is not affected by sampling method. Front. Microbiol..

[bib18] Sbardellati D.L., Fischer A., Cox M.S., Li W., Kalscheur K.F., Suen G. (2020). The bovine epimural microbiota displays compositional and structural heterogeneity across different ruminal locations. J. Dairy Sci..

[bib19] Shen J.S., Chai Z., Song L.J., Liu J.X., Wu Y.M. (2012). Insertion depth of oral stomach tubes may affect the fermentation parameters of ruminal fluid collected in dairy cows. J. Dairy Sci..

[bib20] Song J., Choi H., Jeong J.Y., Lee S., Lee H.J., Baek Y., Ji S.Y., Kim M. (2018). Effects of sampling techniques and sites on rumen microbiome and fermentation parameters in Hanwoo steers. J. Microbiol. Biotechnol..

[bib21] Stewart R.D., Auffret M.D., Warr A., Wiser A.H., Press M.O., Langford K.W., Liachko I., Snelling T.J., Dewhurst R.J., Walker A.W., Roehe R., Watson M. (2018). Assembly of 913 microbial genomes from metagenomic sequencing of the cow rumen. Nat. Commun..

[bib22] Terré M., Castells L., Fàbregas F., Bach A. (2013). Short communication: Comparison of pH, volatile fatty acids, and microbiome of rumen samples from preweaned calved obtained via cannula or stomach tube. J. Dairy Sci..

[bib23] Wallace R.J. (2008). Gut microbiology-broad genetic diversity, yet specific metabolic niches. Animal.

[bib24] Wang M., Wang R., Janssen P.H., Zhang X.M., Sun X.Z., Pacheco D., Tan Z.L. (2016). Sampling procedure for the measurement of dissolved hydrogen and volatile fatty acids in the rumen of dairy cows. J. Anim. Sci..

